# Insulin translates unfavourable lifestyle into obesity

**DOI:** 10.1186/s12916-018-1225-1

**Published:** 2018-12-13

**Authors:** Hubert Kolb, Michael Stumvoll, Werner Kramer, Kerstin Kempf, Stephan Martin

**Affiliations:** 1 Faculty of Medicine, University of Duesseldorf, Moorenstr. 5, 40225 Duesseldorf, Germany; 2 West German Centre of Diabetes and Health, Duesseldorf Catholic Hospital Group, Hohensandweg 37, 40591 Duesseldorf, Germany; 3 Department of Endocrinology and Nephrology, University of Leipzig, Liebigstraße 18, 04103 Leipzig, Germany; 4 Biomedical and Scientific Consulting, 55130 Mainz, Germany

**Keywords:** Insulin, Hyperinsulinaemia, Obesity, Type 2 diabetes mellitus, Lipolysis, Lifestyle

## Abstract

Lifestyle factors conferring increased diabetes risk are associated with elevated basal insulin levels (hyperinsulinaemia). The latter predicts later obesity in children and adolescents.

A causal role of hyperinsulinaemia for adipose tissue growth is probable because pharmacological reduction of insulin secretion lowers body weight in people who are obese. Genetic inactivation of insulin gene alleles in mice also lowers their systemic insulin levels and prevents or ameliorates high-fat diet-induced obesity. Hyperinsulinaemia causes weight gain because of a physiological property of insulin. Insulin levels that are on the high side of normal, or which are slightly elevated, are sufficient to suppress lipolysis and promote lipogenesis in adipocytes. The effect of insulin on glucose transport or hepatic glucose production requires six or two times higher hormone levels, respectively.

It seems justified to suggest a lifestyle that avoids high insulin levels in order to limit anabolic fat tissue activity.

## Background

### Lifestyle, systemic inflammation and hyperinsulinaemia

There are now more overweight people in the world than those who are underweight [1]. There has also been a major increase in the global prevalence of type 2 diabetes mellitus (T2DM) [2]. Lifestyle changes are commonly held responsible for these epidemics, with energy-dense western-style diets and little physical activity being major risk factors. However, other lifestyle factors may confer risks of both obesity and T2DM [3]. All factors are associated with moderately elevated systemic levels of pro-inflammatory mediators, increased fasting levels of insulin and decreased insulin sensitivity. Calorie-rich diets caused postprandial inflammation and hyperinsulinaemia [4, 5]. Continuous excess nutrition more than doubled basal insulin levels within 4 days, but did not cause elevated basal glucose levels [6]. Increased physical activity or reallocation of sedentary time to physical activity lowers fasting insulin concentrations and the level of systemic inflammation [7]. Conversely, short-term decreased physical activity, with increased sedentary behaviour, increased whole-body insulin resistance [8]. In an experimental setting, exposure to road traffic-associated fine particulate matter was associated with higher levels of inflammatory markers, insulin and insulin resistance [9]. Sleep deprivation, even for one night, increases systemic insulin resistance [10, 11] and is accompanied by systemic inflammation [12] (Table 1). Although only studied using epidemiological approaches, a positive association has been observed between smoking, depression, stress or low socioeconomic status and inflammation or hyperinsulinaemia/insulin resistance [13–18].

**Table 1:** Association of obesity risk factors with low-grade systemic inflammation and hyperinsulinaemia

Lifestyle factor	Inflammation	Hyperinsulinaemia/ insulin resistance	References
Calorie-rich diets	Yes ^a^	Yes ^a^	[4, 5, 114]
Sedentary time	Yes ^a^	Yes ^a^	[7, 8, 115]
Road traffic	Yes ^a^	Yes ^a^	[9, 116, 117]
Sleep deprivation	Yes ^a^	Yes ^a^	[10–12]
Smoking	Yes	Yes	[14, 15]
Depression, stress	Yes	Yes	[13, 16]
Low socioeconomic status	Yes	Yes	[17, 18]

^a^ Randomised controlled trials indicate causal relationship

Largely independent of obesity status, inflammation appears to be a rapid response to an unfavourable lifestyle [19, 20] and may be responsible for metabolic deterioration. For instance, low levels of pro-inflammatory cytokines such as interleukin-1 increased insulin secretion by ß-cells [21]. Pro-inflammatory cytokines interfere with insulin signalling; for example, the induction of IĸB kinase ß, which phosphorylates serine residues of IRS-1 and thereby interferes with insulin signalling [22, 23].

Several other pathways may be involved in promoting obesity by environmental/lifestyle factors; e.g., the hypothalamic–pituitary–adrenal axis, because increased cortisol exposure enhances fat accumulation in visceral depots [24]. Hypothalamic circuits contribute to appetite regulation and energy homeostasis [25]. Recently, glial and endothelial cells have reportedly contributed to metabolic disorders and obesity [26, 27]. Genetic studies confirm the association between neurodevelopmental loci and obesity [28]. Another player is the microbiota [29]. Lipid fluxes and the liver are expected to affect the development of hepatosteatosis and obesity [30].

Independent of the initial effect of lifestyle factors, the pathway to obesity requires hyperinsulinaemia as a critical mediator in translating an unfavourable lifestyle into body weight gain.

## Main text

### Hyperinsulinaemia versus obesity: epidemiological findings

Prospective studies exploring whether hyperinsulinaemia precedes and predicts later obesity have mostly been conducted in children and adolescents. Several studies found fasting hyperinsulinaemia and insulin resistance to be risk factors for weight gain in later years [31–35]. In a recent cohort that included 39% obese children, fasting hyperinsulinaemia did not predict change in body mass index (BMI), except for more weight gain in obese children [36]. Studies in adults do not offer consistent results. Fasting hyperinsulinaemia predicted weight gain in postmenopausal women, except in the most obese [37]. By contrast, high fasting insulin levels were associated with lower rates of weight gain in cohorts with a mean BMI of 26 kg/m^2^ [38, 39] and in obese people [40, 41].

These results indicate that insulin levels may predict obesity in children and adolescents. Conclusions drawn from adult studies are less clear. However, these observational studies did not document and control for all lifestyle-dependent factors of obesity risk, all of which impact insulin secretion (Table 1). Only one study analysed dietary intake and an interaction was found between fasting insulin, total calories consumed, and fat percentage in predicting weight gain [42].

### Hyperinsulinaemia versus obesity: intervention trials

A more direct approach for assessing the role of fasting (and diurnal) levels of insulin in weight gain includes interventions targeting insulin secretion. Insulin secretion can be partially inhibited with the potent ß-cell K_ATP_ channel opener diazoxide [43]. In a randomised controlled trial, diazoxide in conjunction with a hypocaloric diet for 8 weeks led to greater weight loss in obese people than those in the control group treated by diet alone [44]. In the diazoxide group, insulin levels decreased by 36% (fasting) and ~55% (post intravenous glucose) without differences in blood glucose levels compared with the control. In a similar trial, diazoxide did not induce more weight loss than the hypocaloric diet alone in the control group [45]. Unfortunately, the baseline fasting insulin levels in this study were significantly higher in the diazoxide group (by 32%) compared to the diet-alone group and the decrease in insulin secretion was not different between the diazoxide and control groups after 8 weeks of treatment. Taken together, body weight was reduced in all trials in which diazoxide achieved a lowering of basal and postchallenge blood glucose levels.

Insulin secretion can also be lowered by the long-acting somatostatin analogue octreotide. This peptide binds with high affinity to somatostatin receptors 5 and 2, effectively suppressing hormone production in ß-cells and several other endocrine cells, such as those in the pituitary or gut [43]. Severely obese adults were treated for 24 weeks with a dose of octreotide that significantly suppressed insulin production (and possibly other hormones). Stimulated insulin indices were reduced by 57% and there was significant weight loss (–3.5 kg/–2.8%) [46]. Secondary analyses showed that insulin was not significantly decreased in the subgroup with weight gain. By contrast, the remaining subgroup exhibited both a decrease in insulin secretion and substantial weight loss (–5.6 kg). In a subsequent similar study with three doses of long-acting octreotide and a randomised placebo control, the two higher doses caused significant weight loss (–2.1 kg/–1.9%) compared with the control group (–0.1 kg) [47]. As in the diazoxide trials, body weight reduction was only observed in association with lowered insulin levels.

Both compounds used to decrease insulin secretion have other pharmacological effects that may contribute to the weight loss observed. Diazoxide causes smooth muscle relaxation and fluid retention, while octreotide has a low risk of cardiac, hepatic and renal toxicity [48, 49]. However, the two drugs represent quite different pharmacological approaches, which share an insulin lowering effect but not adverse effects. Body weight reduction was only noted in conjunction with decreased insulin secretion.

In T2DM, treatment with exogenous insulin increases systemic insulin levels and this may support fat tissue growth [50]. The heterogeneous nature of T2DM means it is difficult to draw conclusions about the role of insulin or hyperinsulinaemia in a healthy metabolic state. Therefore, insulin therapy in type 1 or type 2 diabetes is not discussed here.

Increasing insulin concentrations in the brain appears to have opposite effects. Cerebral insulin is an anorexic hormone, but its actions are impaired in obese people because of brain insulin resistance [51]. Intranasal insulin delivery suppresses food intake and enhances postprandial thermogenesis, with concurrent lowering of postprandial systemic insulin levels [52, 53].

### Hyperinsulinaemia versus obesity: genetic studies

Currently, selective lowering of circulating insulin levels without pharmacological effects in other organs can only be achieved by genetically manipulation. Mice harbour two insulin genes, *Ins1* and *Ins2.* The tissue distribution pattern of *Ins2* resembles that of the human insulin gene, while *Ins1* is expressed in ß-cells only. Glucose homeostasis remains normal after ablation of either insulin gene. After disrupting *Ins2* and one allele of *Ins1*, fasting insulin was substantially reduced (>50%) without persistent effects on glucose homeostasis [54]. When fed an obesogenic high-fat diet, these mice did not become obese, did not develop hepatic steatosis and increased energy expenditure in association with browning of white adipose tissue. In a second study, female mice with a deleted *Ins1* gene and only one intact *Ins2* allele exhibited lower insulin levels, but only during the first 6 months. However, this was sufficient to protect them from high-fat diet-induced obesity over one year of observation [55]. In a recent study using mice expressing only *Ins2* (both alleles), mice exhibited compensatory high insulin production from *Ins2* genes and developed obesity on a high fat diet. The *Ins2* gene had been modified to allow reduction of the insulin gene dosage by the Cre-loxP system. Partial ablation of *Ins2* alleles in adult obese mice led to significant weight loss, with a specific effect on visceral adipose tissue [56]. A moderate reduction in fasting insulin levels was seen (30%) and did not affect glucose tolerance, insulin sensitivity, glucose-induced insulin secretion or body growth under a low or moderate fat diet. There were no differences in the levels of several other circulating hormones, including leptin, resistin, ghrelin, GIP, GLP-1, IL-6, and PYY.

**Fig. 1:**
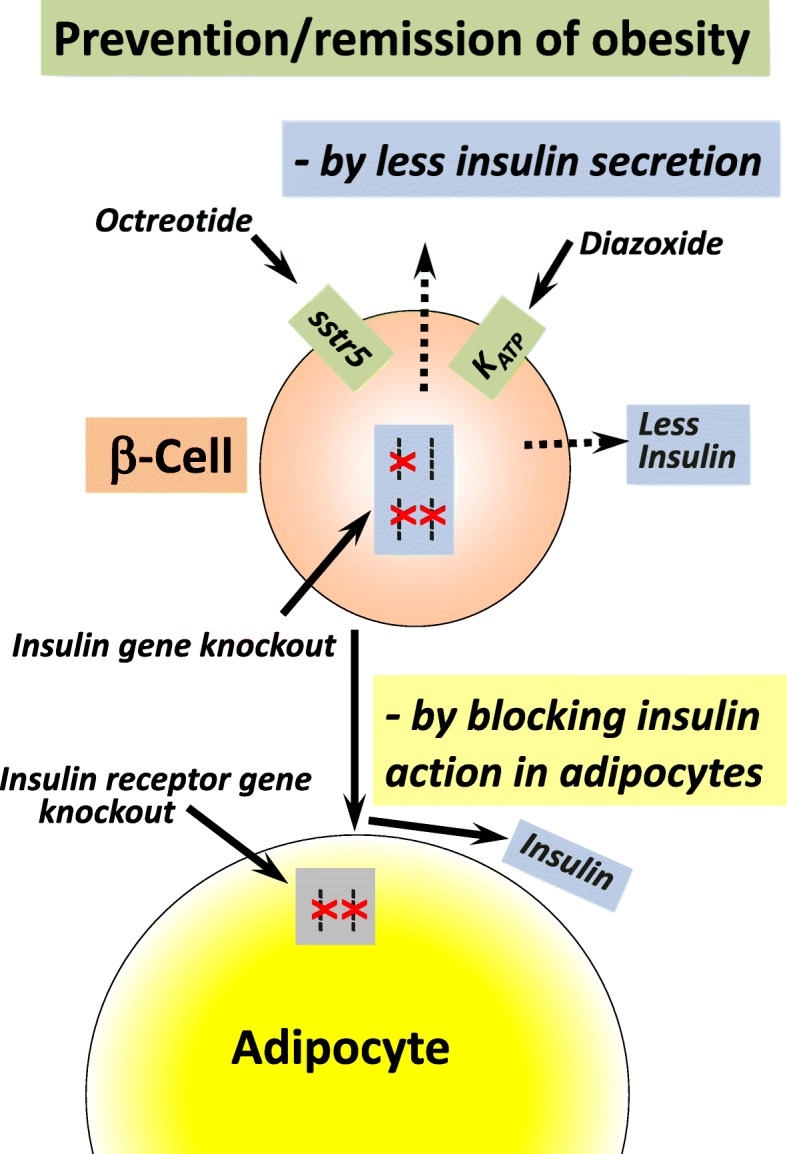
Prevention/remission of obesity by targeting insulin or insulin action. In people who are obese, lowering insulin secretion by treatment with the ß-cell K_ATP_ channel opener diazoxide, or the long-acting somatostatin analogue octreotide, caused significant weight loss compared with the control group

The role of insulin in adipose tissue growth was also tested by selectively disrupting the insulin receptor gene in fat cells of white and brown adipose tissue. Such mice grew normally and their glucose tolerance was not different from control littermates. Basal glucose uptake into adipocytes was unchanged, but insulin-stimulated glucose uptake reduced by ~90%. Mice with such selective insulin resistance of adipose tissue had low fat mass and were protected from age-related obesity [57]. In summary, four different approaches to lowering insulin secretion had the same consequence: prevention or remission of obesity (Fig. 1).

Mutations of the human insulin gene have been described that affect insulin secretion. However, in all cases, insulin secretion was impaired to an extent that resulted in increased fasting glucose levels or diabetes [58–60].

### Hyperinsulinaemia: old findings reappraised

Weight gain leading to an overweight BMI is usually caused by fat tissue growth rather than muscle. Ectopic storage of triglycerides in many other cell types, including liver and muscle cells, also contributes [61].

Insulin’s lipogenic activity has been well studied and and is identical in concentration dependence to its inhibitory action on lipolysis; i.e., signalling through the insulin receptor on adipocytes causes simultaneous inhibition of lipolysis and storage of triglycerides [62]. One observation, first made in the 1980s, is that lower concentrations of insulin are required to inhibit lipolysis in adipocytes than are needed to promote glucose influx into peripheral tissue. In hyperinsulinaemic–euglycaemic studies, the concentrations of plasma insulin required to lower plasma levels of the products of triglyceride metabolism by 50% were 42–120 pmol/l (mean = 78 pmol/l), in non-obese subjects [63–69]. The mean fasting insulin level of all study groups combined was 48 pmol/l; i.e., people with fasting insulin levels above the mean had substantially inhibited lipolysis. In another hyperinsulinaemic–euglycaemic study published in 1999, the inhibitory action of systemic insulin was similar between adipose and muscle tissue. Increasing insulin concentrations from 50 to 63 pmol/l already significantly inhibited glycerol release by around 20% [70].

**Fig. 2:**
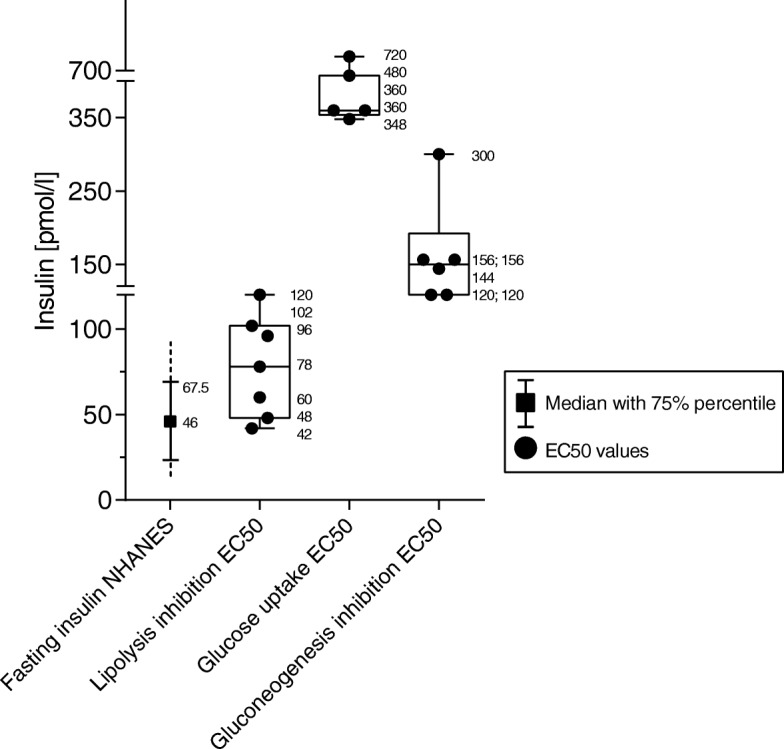
Fasting insulin levels compared with EC50 values for lipolysis inhibition, glucose uptake induction or gluconeogenesis inhibition. Fasting insulin data are taken from the National Health and Nutrition Examination Survey (NHANES) 1986–1994 [71]. Numbers in rectangular boxes indicate mean insulin concentration for 50% effect (EC50) on the stimulation of peripheral glucose uptake, as determined in different studies of non-obese adults. Mean insulin EC50 values for lipolysis inhibition were taken from [63, 64, 66–69, 113]; mean EC50 values for the stimulation of glucose uptake were taken from [64, 65, 67, 74, 75]; and mean EC50 values for the inhibition of gluconeogenesis were taken from [63–65, 67, 74]

Since most of these studies were North American, we compared figures with the normal range of fasting serum insulin levels of the representative National Health and Nutrition Examination Survey 1988–1994. Geometric mean fasting serum insulin levels for non-obese nondiabetic people were ~46 pmol/l [71]. This suggests that more than half of the adult non-obese population in the USA had fasting insulin concentrations in the range required to inhibit ≥ 50% of lipolysis (Fig. 2). At the individual level, the relationship between insulin concentrations and lipolysis will be difficult to predict because of the substantial variation in fasting insulin concentrations (or insulin resistance) between non-obese people with varying genetic backgrounds and lifestyles [72]. Increased insulin resistance may attenuate increased lipolysis inhibition in people with higher fasting insulin levels. Such an interrelationship indeed exists, but two-thirds of the individual variation of insulin resistance cannot be explained by fasting insulin levels [73].

More important is the observation that stimulating glucose uptake by insulin requires much higher hormone concentrations than is needed to inhibit lipolysis, even in the same individual. In five clamp studies, the mean insulin concentrations required to have a 50% effect (EC50) on the stimulation of peripheral glucose uptake were ~720, 480, 348, 360 and 360 pmol/l (mean = 454 pmol/l) [64, 65, 67, 74, 75]. Half maximal stimulation of glucose uptake required an insulin concentration that was about six times higher compared with that required for 50% inhibition of lipolysis (Fig. 2).

Suppression of hepatic glucose production also required higher insulin concentrations than lipolysis inhibition – again, in part measured in the same individuals. EC50 values for insulin were ~300, 156, 156, 144, 120, 120 pmol/l (mean = 170 pmol/l) [63–65, 67, 74]. Suppression of hepatic glucose output by 50% thus required more than twice the insulin concentration needed for half maximal inhibition of lipolysis (Fig. 2). To obtain the latter findings, insulin was infused at a peripheral vein to deliver a steady concentration of exogenous insulin to the liver via the arterial circulation. Usually, insulin is released from ß-cells in discrete pulses, about every 5 minutes, with an amplitude of 0.5–1 nmol/l insulin in the fasted state and up to 5 nmol/l after a meal [76]. Of these high amounts of insulin, 50–80% is taken up by hepatocytes and does not therefore reach the peripheral circulation. Since hepatocytes can modulate the extent of insulin clearance, peripheral insulin levels are not only determined by ß-cell function [76, 77].

Taken together, evidence is compelling that insulin levels on the high side of normal, or which are slightly elevated, substantially inhibit lipolysis in the absence of relevant insulin actions on hepatic glucose production or on glucose transport into muscle. Even in high fasting plasma insulin concentrations, lipolysis inhibition is the most sensitive response to insulin (Fig. 2). Since lipolysis inhibition is equivalent to lipogenesis promotion, the effect of insulin on adipocytes is probably responsible for the observed association between hyperinsulinaemia and incident obesity. It also fits with the anti-obesity effects of lowering insulin levels with diazoxide or octreotide, and with the prevention or remission of obesity with genetic downregulation of insulin production or insulin receptor expression on adipocytes in mice. Studies in rodents also suggest that reducing circulating insulin levels by inactivating insulin genes or with diazoxide increases the basal metabolic rate by enhanced heat production from mitochondria during fat oxidation by uncoupling protein 1 [54, 78].

### Hyperinsulinaemia: the bigger picture

Inhibition of lipolysis/promotion of lipogenesis, hepatic gluconeogenesis and glucose uptake into insulin-sensitive cells via upregulation of glucose transporter GLUT4 all require different levels of insulin to signal effectively via their receptors. Currently, the most probable explanation is the activation of different insulin signalling pathways in the different tissues, such as the PI3K-Akt pathway versus the MAP kinase pathway [79]. Insulin stimulates glucose transport via the canonical PI3K-Akt pathway, whereas lipolysis is suppressed via Akt-independent suppression of protein kinase A [80]. A further concept is that of selective insulin resistance. Indeed, insulin resistance affects glucose uptake but does not interfere with ChREBP-ß-dependent de novo lipogenesis [79, 81].

It is not currently possible to disentangle the insulin-dependent regulatory network controlling body weight and weight increase. Insulin modifies its own activity [82] and interacts with other regulatory factors, such as other hormones, neuronal activity or gut function [83–85]. Genetic components, such as putative thrifty genes and DNA sequences associated with obesity risk, add further complexity [86, 87]. It is therefore almost surprising that modulation of the systemic level of a single hormone, insulin, has such profound consequences on the risk of becoming obese. Insulin is our dominant anabolic hormone and, during an anabolic state of metabolism, cell stress is increased [20]. Insulin resistance is therefore considered a physiological defence to limit damage [88]. Low insulin levels extend the lifespan – at least in mice, possibly because of lower oxidative stress [89–91]. This effect was seen in the absence of altered IGF-1 levels and was associated with lower fasting glucose and improved insulin sensitivity.

The inverse association between insulin-mediated lipolysis and lipogenesis in adipocytes [62] means that decreased adipose tissue growth is accompanied by increased release of non-esterified free fatty acids (FFAs) from adipocyte triglycerides because of increased lipolysis. Systemic FFAs mostly come from upper body subcutaneous fat and do not reflect visceral adiposity [92]. Individual fasting FFA concentrations vary substantially – even if measured on consecutive days (coefficient of variation, 45%, versus 4.8% for fasting glucose) [93]. One reason may be that the half-life of FFAs in the circulation is only 2–4 minutes [94].

The epidemiological association between increased FFA levels after an overnight fast with metabolic and cardiovascular outcomes is not convincing, because the opposite has also been reported [94–96]. Women have FFA levels that are approximately 20% higher than men, yet they have similar insulin sensitivity [92, 94]. Fasting FFA concentrations were not associated with several measures of insulin resistance or with liver fat accumulation [97].

In lean people, mean overnight fasting FFA concentrations ranges between 300 and 600 μmol/l [94]. Obese people with manifold higher fat mass exhibit marginally higher FFAs (difference ~70 μmol/l, mean of 43 studies) in the circulation [94]. This indicates that fat tissue releases fewer FFAs in obesity, attributed to downregulation of enzymes involved in the breakdown of triglycerides [98]. By contrast, FFA levels increased by ~60% after 24 hours of severe energy restriction (2.3 MJ) and reached around 1300 μmol/l after a 72-hour fast [94, 99]. Severe energy restriction of a similar magnitude (2.5 MJ/day) in T2DM patients has reportedly substantially improved metabolic health and even reverted clinical T2DM [100]. As expected, there was a decrease in plasma insulin levels by approximately one-third, reaching the level of non-diabetic controls, and a concomitant increase in systemic FFAs by ~40%. However, elevated FFA concentrations reverted to baseline levels within 8 weeks. Similarly, bariatric surgery lowered fasting insulin levels, accompanied by an increase of FFA concentrations, but FFA levels returned to baseline or lower after several months [101]. These findings indicate that humans adapt to lower insulin levels by normalising initially increased FFA levels.

## Conclusions

Barbara Corkey introduced the concept of hyperinsulinaemia as a risk factor for obesity [102]. She suggested that environmental agents, such as food additives, toxins or excess iron, which have entered the food chain since 1980, might cause insulin hypersecretion [102, 103]. We report here that all lifestyle characteristics known to confer a risk of obesity are associated with hyperinsulinaemia. Any relationship between unfavourable lifestyle factors and high consumption of food additives or toxins at a global level remains unknown. Here, we suggest a mechanism for the association between hyperinsulinaemia and obesity, based on work mostly published in the 1980s. These studies concur in that much lower concentrations of insulin are required to inhibit lipolysis compared with gluconeogenesis or the promotion of glucose uptake (Fig. 2).

Interestingly, fasting insulin levels were much lower in lean vegetarians (mean = 30 pmol/l) than in a lean case control group with similar energy and major nutrient intake (mean = 44 pmol/l) and there was only a minor difference in fasting glucose values (means = 4.47 versus 4.71 mmol/l) [104]. Although insulin acts in virtually all tissues of the body, the low insulin levels of vegetarians are apparently sufficient to maintain the hormonal effects of insulin in the body. The very low basal insulin concentrations in vegetarians indicate that fasting insulin levels are modified by type of diet. The strongest effects are seen in people who follow very low calorie diets [100], intermittent fasting [105], or undergo bariatric surgery [106]. Dietary interventions are complicated by a diurnal pattern of insulin resistance, being lowest in the morning [107, 108]. Therefore, skipping breakfast has less favourable consequences than skipping dinner [109]. Physical exercise also reduces fasting insulin levels [7] (independently of weight change because it can be observed after a single exercise session) [110].

It may be insufficient to recognise prolonged hyperinsulinaemia by monitoring diurnal glucose levels. For instance, people with higher fasting insulin levels but normal glycaemia respond with higher postprandial insulin secretion than people with low basal insulin [111]. In healthy adults, oral glucose tolerance was not affected by the level of physical activity on the preceding day, but serum insulin levels during the glucose tolerance test were lower after high physical activity [112].

Taken together, the data presented justify the recommendation of a lifestyle that avoids high insulin levels for much of the day to limit the period of anabolic fat tissue activity (Box 1).

## Box 1: Key points

• All known lifestyle-dependent obesity risk factors are associated with, or give rise to, hyperinsulinaemia.

• Insulin levels that are on the high side of normal, or that are slightly elevated, predict later obesity in children and adolescents but not in adults.

• Pharmacological lowering of insulin secretion by diazoxide or octreotide causes weight loss.

• In mice, genetic lowering of insulin levels or selective genetic disruption of the insulin receptor in adipocytes causes prevention or remission of obesity.

• Lipolysis inhibition is the most sensitive metabolic action of insulin. Consequently, fasting insulin levels that are on the high side of normal, or that are slightly elevated, are sufficient to substantially inhibit lipolysis and the promotion of concomitant lipogenesis in adipocytes.

• Insulin concentrations that are six times higher than normal are required to stimulate glucose uptake and two times higher than normal to inhibit gluconeogenesis.

• It seems justified to suggest a lifestyle that avoids high insulin levels for much of the day to limit the period of anabolic fat tissue activity. Appropriate measures include low calorie diets, intermittent fasting or physical activity.

## Abbreviations

BMI: Body mass index
EC50: Mean insulin concentration for 50% effect
FFAs: Free fatty acids
T2DM: Type 2 diabetes mellitus
